# Health Equity Intervention for Youth with Type 1 Diabetes and High Social Risk

**DOI:** 10.3390/children12020200

**Published:** 2025-02-08

**Authors:** Stephanie M. Stover-Kempers, Kristen A. Torres, Samantha A. Barry-Menkhaus, Celeste Jenisch, Kim Spiro, Michael A. Harris, David V. Wagner

**Affiliations:** Department of Pediatrics, Oregon Health & Science University, Portland, OR 97239, USA; stovers@ohsu.edu (S.M.S.-K.); torreskr@ohsu.edu (K.A.T.); samantha.a.barry@gmail.com (S.A.B.-M.); celeste.jenisch@gmail.com (C.J.); spiro@ohsu.edu (K.S.); harrismi@ohsu.edu (M.A.H.)

**Keywords:** diabetes, comparison group, retrospective cohort, matched pair, behavioral health, health equity, social risk, admissions, glycemia, health disparities

## Abstract

Background/Objectives: Youth with type 1 diabetes (T1D) who experience avoidable complications often have dangerously high and consistently elevated HbA1c values. Novel Interventions in Children’s Healthcare (NICH), a program designed to effectively intervene with this population, has demonstrated success with reducing avoidable complications and improving HbA1c in these youth. However, prior examinations of program outcomes have not included a comparison group. This is the first study to compare electronic health record (EHR) outcomes (i.e., HbA1c values, hospital utilization) of NICH youth to a comparison group. Methods: Youth with T1D and avoidable complications were referred to NICH (*n* = 101; NICH = 40; comparison = 61) from the Pacific Northwest region of the United States. Retrospective EHR review included one year prior to and two years post NICH referral. Outcomes included hospitalization utilization and HbA1c values. There were no significant demographic differences between NICH and unserved youth (M age = 14.05 years; 50% female). Results: Within-group analyses revealed that NICH youth demonstrated a significant reduction in mean (M) admissions from one year prior to two years post-referral (M = 1.55 to M = 0.99; *p* = 0.011) as well as reduced HbA1c values from pre-referral to one year post-referral (M = 11.64%; 287 mg/dL; 15.9 mmol/L to M = 10.87; 265 mg/dL; 14.7 mmol/L; (*p* = 0.006)). Between-group analyses revealed NICH youth had lower proportions of individuals with an HbA1c over 10% (240 mg/dL; 13.3 mmol/L) (*p* = 0.03) compared to comparison group youth at one year post-referral. ANOVA analyses showed a significant reduction in admissions in linear interaction F (1,95) = 4.036, (*p* = 0.047), indicating that NICH youth demonstrated a significantly greater reduction in admissions over time compared to comparison youth. Conclusions: This study was the first to compare the health outcomes of NICH youth to a comparison group. NICH youth demonstrated significant reductions in admissions and HbA1c values over time.

## 1. Introduction

Many youths with type 1 diabetes (T1D) experience challenges in diabetes self-management, resulting in higher HbA1c values, elevated diabetes distress, insufficient access to diabetes technology and specialty care, as well as an increased risk of short- and long-term complications [1,2]. These youth may experience avoidable complications and most certainly experience cumulative damage from chronically high HbA1c levels [3]. Youth with multiple complex social risks are likely particularly vulnerable to experiencing challenges with self-management of T1D, highlighting the importance of developing and evaluating interventions for this population.

Some outpatient and telehealth interventions for youth with T1D have been efficacious in improving self-management. These include services such as behavioral family systems therapy, cognitive behavioral therapy, crisis lines, telehealth, and care coordination efforts [4,5]. However, these interventions often rely on families being able to access and consistently engage in care. Barriers to treatment access and challenges with engagement among youth and families from under-resourced groups can result in reduced intervention effectiveness and chronically elevated HbA1c values [6]. Existing interventions for youth with T1D tend to be confined to standard clinic hours, require reliable transportation, the ability for a parent or guardian to take off work, and/or a stable internet connection, resulting in these primarily being accessed and effective for those with the greater resources [4,7].

In addition to most interventions not being built for and accessible to youth with T1D at greatest risk of complications, findings demonstrating the efficacy of these interventions are often not generalizable to youth from families experiencing high social needs. Even when meeting inclusion criteria, these youth and families are historically difficult to recruit and retain in intervention studies. In addition to financial and logistical barriers described above, families who have been marginalized have an earned distrust of formal systems and related research. Moreover, those experiencing homelessness, domestic violence, or other unstable living conditions may have understandable difficulty prioritizing or committing to participation. Furthermore, many clinical trials have strict eligibility criteria, geographic barriers, and/or complex protocols that may unintentionally exclude people with social needs. These factors also create barriers to including comparison groups to assess the effectiveness of interventions targeted towards these families [8]. Youth with a combination of T1D and high social needs experience a disproportionate risk of complications [9]. Excluding such individuals due to challenges with enrolling or comorbidities that result in ineligibility can do a disservice to populations who are in greatest need of intervention [10].

To address this gap in the continuum of care, Novel Interventions in Children’s Healthcare (NICH) was developed to engage and effectively intervene with youth who experience significant social challenges that make accessing and benefiting from standard medical and/or behavioral health services near to impossible [11,12]. NICH serves youth with chronic and/or complex health conditions in their homes and communities and has evidenced success across multiple regions and healthcare systems in reducing health disparities in patients with T1D. Despite these youth experiencing high social risk (e.g., housing and food insecurity, and exposure to violence), NICH engagement has been associated with clinically and statistically significant improvements in HbA1c and fewer acute complications (emergency (ED) visits and admissions) related to diabetes care [12,13,14]. Despite evidence of positive associations between NICH involvement and health as well as cost outcomes, youth outcomes without the program are less understood [15]. This study is designed to address potential retrospective cohort design limitations by comparing youth with T1D who received NICH to those with similar risk profiles who were referred but never served by the NICH program [16,17,18,19]. Studies that have utilized similar retrospective cohort designs (e.g., [17,19]) note the utility of this design as it eliminates the ethical concern of withholding treatment from a comparison group, an issue that is especially prominent when treating high-risk populations. This design may also reduce bias in analysis as it utilizes previously collected data that was gathered without prior knowledge of the study [16,17,20].

The objectives of this present study are (1) to examine changes in health outcomes (i.e., HbA1c, acute complications) of youth who received NICH and (2) to compare changes in health outcomes to similar youth who were referred but not served.

## 2. Materials and Methods

**Figure 1:**
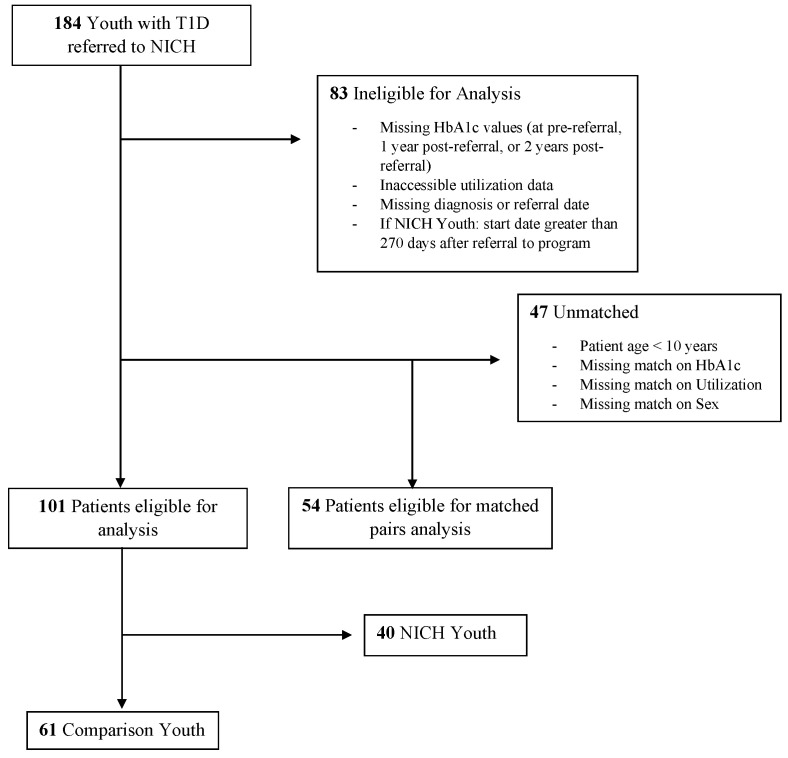
CONSORT diagram.

Inclusion criteria were the following: (1) youth aged 18 or younger, (2) a diagnosis of T1D, (3) elevated HbA1c and/or presence of T1D-related complications in the prior year, (4) and referral to the NICH program by a medical provider. Subjects with missing data at any required timepoint or within any outcome category (e.g., admitted days) were omitted from respective analyses. Many youth referred to NICH experience complex co-occurring or secondary diagnoses (e.g., celiac disease, Hashimoto’s, and pancreatitis). Youth were only excluded from analyses if T1D was secondary to their main referral concern (e.g., acute lymphoblastic lymphoma and cystic fibrosis) such that it would render their T1D experience and treatment drastically different from that of their peers. EHR review included collecting HbA1c values, hospital admissions, days admitted, and ED visits over a 3-year period (one year pre-referral, one year post-referral, two years post-referral). HbA1c values were identified through chart review. If more than one value existed during a time range for analysis (e.g., the year prior to referral), the value collected closest to the end of the time period was selected. Timepoints for analysis were based on NICH referral dates rather than NICH service start dates, as unserved youth do not have a NICH start date for comparison. HbA1c values were excluded from analyses if they were not recorded within the respective predetermined timeframes for analyses. NICH youth with a gap of 270 days or more between referral and start dates were excluded from analyses due to concern that findings were unlikely to represent NICH treatment. (See Consort Figure 1 for a full breakdown). Although diabetes technology was not a specific focus of this study, it is noteworthy that in the year prior to referral, use of a continuous glucose monitor (CGM) and/or an insulin pump was low across both groups. Among NICH Youth, 7.5% utilized insulin pumps and 2.5% utilized CGMs, while comparison youth demonstrated 5.7% with insulin pumps and 1.9% with CGMs. Closed-loop hybrid technology was not utilized by youth in either group.

Comparison Group: If youth were referred to but not able to access NICH in this study, it was due to lack of insurance company approval or residing in a community that lacked access to NICH services. Youth in this group (i.e., comparison youth) typically accessed a multi-specialty team that included pediatric endocrinology and nursing, diabetes educators, dieticians, social workers, and pediatric psychologists with expertise in diabetes. Many youth also received additional behavioral health services in their community as well as case management and/or care coordination through their insurer.

NICH Program Description: NICH is an intensive home- and community-based treatment service for youth with high medical and psychosocial complications and their families [1]. NICH services are tailored to each family’s unique situation, needs, and goals. Program goals include both individualized goals for each family (i.e., addressing conflict within the family related to disease management, personalizing medication regimen reminders, engaging in relevant resources within the family’s local community) and common goals including improved youth health, reduction of provider burnout, improved youth experience of care, lower healthcare costs, and improved health equity as reflected by the Institute for Healthcare Improvement’s Quintuple Aim [21]. Requirements for NICH program approval include presence of a complex and/or chronic medical condition (e.g., T1D, cystic fibrosis, epilepsy, chronic pain, and renal failure) and significant psychosocial vulnerabilities (e.g., co-occurring parent or child mental health diagnoses, unemployment or underemployment, houselessness, food insecurities, cultural and/or language barriers, involvement with child protective services (CPS), and trauma). Additionally, over 95% of families and youth involved in the NICH program are living in poverty and experience various related risks. Bronfenbrenner’s theory of social ecology is a cornerstone of the NICH program, which posits that youth outcomes are influenced by the systems and relationships in which youth are embedded [12].

NICH is provided by “interventionists” with associate’s, bachelor’s, or master’s degrees, and their prior training often includes public health, social work, family studies, and nursing, as well as other fields not directly related to health and human services. Interventionists deliver services to youth and their families in settings that are easy to access and where challenges in disease management are likely to occur for the family, including home, school, clinics, and other regularly utilized community spaces. Interventionists are flexibly available (e.g., evenings and weekends) to families in order to meet urgent needs after standard business hours as well as to maximize opportunities to serve NICH participants on a schedule that aligns with participants’ daily lives, with over 50% of interactions occurring outside of standard business hours [1]. To counteract the risk of burnout, caseloads are kept relatively small (e.g., 8 families per interventionist) compared to other programs intended to serve similar populations. In addition to frequent in-person engagement, communication via text message and video chat is utilized in order to broaden opportunities for connection with NICH youth and families. Interventionists typically meet in person with families weekly, outreach to families with at least five text messages per day, and provide approximately five hours per week of service delivery-related efforts (e.g., travel, consultation, and service provision) over the course of a year of services [1].

NICH interventionists coordinate services across multiple systems within the youth’s environment, such as schools, CPS, and other community services (e.g., mental health providers, employers, and housing agencies), and the medical teams directly involved with the youth and family. Interventionists also assist families with accessing pertinent resources in their respective local communities, such as food banks and rent assistance. Acknowledging differences in need and availability of each family, frequency, duration, and setting of interventionist contact varies and is individually tailored to best meet families’ unique needs. A critical component of interventionists’ services includes direct work with individuals involved in the patient’s care, including specialists (e.g., endocrinologists and diabetes educators), primary care physicians, school nurses, and clinic care managers. Interventionists collaborate closely with medical providers, providing insights into the role of social risks impacting patient engagement, identifying families’ communication preferences, and tailoring medical education and other communication efforts to align with the unique needs of the youth and families served in order to support family and medical team goals (e.g., improved participation in disease self-management, alignment with dietary recommendations, and increased outpatient attendance). Referred youth with T1D will often specifically receive coaching on skills such as checking blood glucose levels, carbohydrate corrections, and consistency with dosing of long-acting insulin. Although NICH interventionists are not mental health providers, interventionists assist eligible families with accessing mental health resources and support engagement with recommendations given by qualified mental health providers, with consultation from licensed medical and behavioral health providers to support these skill-building efforts. Discharge from the program is based on youth, caregivers’, and medical team determination that NICH service goals have been met. Common goals include evidence that barriers to accessing medical care have been addressed, lab values or adherence data illustrate clinically meaningful health improvements, engagement and relationships with medical providers shows improvement, and families have demonstrated that they can continue to manage the youth’s medical condition without direct support from NICH. The standard length of NICH services is one year, and interventionists gradually titrate the frequency and duration of NICH services and meetings as NICH youth approach the end of their year of services. However, in the event that significant patient and/or family needs remain, NICH may pursue an extension of services depending on patient and family interest and insurance authorization. Harris et al. have previously described the NICH intervention with explicit case examples [11,12].

Data Analytic Plan: Data characteristics (e.g., skewness and kurtosis) were first examined to determine appropriate analyses. To examine differences in variables (e.g., ED visits, gender, and HbA1c) between groups prior to referral, we used a combination of chi square, independent *t*-tests, and non-parametric *t*-tests (Mann–Whitney tests) to account for the violations of normality. To assess for within-group differences over time, we used Wilcoxon nonparametric *t*-tests. To assess between-group differences, repeated measures ANOVAs were conducted to examine changes in utilization and HbA1c over time, including Medicaid as a covariate. All analyses were conducted using SPSS version 30. Missing data was minimal, and cases were removed from analyses if they had missing data.

## 3. Results

### 3.1. Demographics

Eligible youth (*n* = 101) included 40 who received NICH and 61 comparison youth who were referred to NICH but never enrolled in the program. For full demographic data, see Table 1. Fifty percent of youth identified as female; 11.9% of youth identified as Hispanic/Latino, and 86.1% identified as white. Youth in NICH were significantly more likely to be on Medicaid (*p* < 0.05) compared to comparison youth.

### 3.2. Health Outcome Characteristics

Mean (henceforth described as “M”) and frequency of health outcomes in the NICH group versus the comparison group as well as a matched comparison group, as can be seen in Table 1. When examining data distribution across all three timepoints using Shapiro–Wilk test, ED visits (*p* < 0.001), admissions (*p* < 0.001), days admitted (*p* < 0.001), and HbA1c (pre-referral *p* < 0.001) one year post-referral (*p* = 0.026) and two years post-referral (*p* = 0.020) were all characterized as not normally distributed. When examining pre-referral outcomes, youth who received NICH had significantly more admissions (M = 2.25; *p* = 0.33) and ED visits (M = 1.55; *p* = 0.011) in the year prior to referral when compared to Comparison youth (M = 1.26 and M = 0.89, respectively). Within those who received NICH, the number of days on the waitlist (which occurred in the first year post-referral) was significantly associated with acute utilization (i.e., ED visits: *p* < 0.05) in the first year post-referral, indicating that outcomes during this time period may not be wholly representative of NICH involvement. As such, all analyses included both a version examining all data points (3 timepoints) and a version just comparing the year prior to referral to two years post (2 timepoints).

#### 3.2.1. Within-Group Analyses

**Figure 2:**
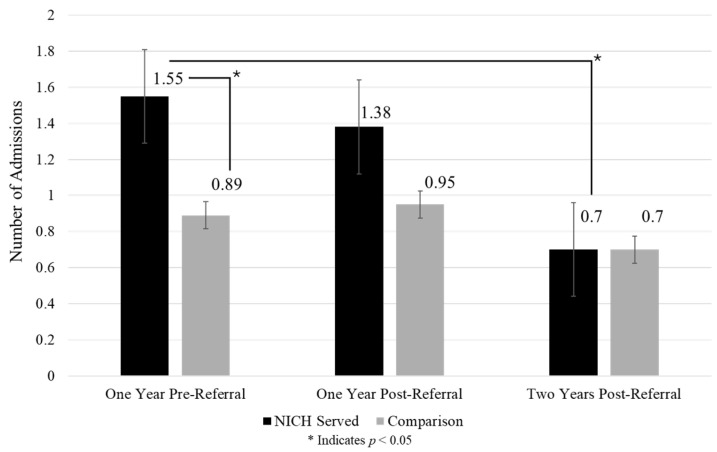
Admissions across timepoints between groups.

**Figure 3:**
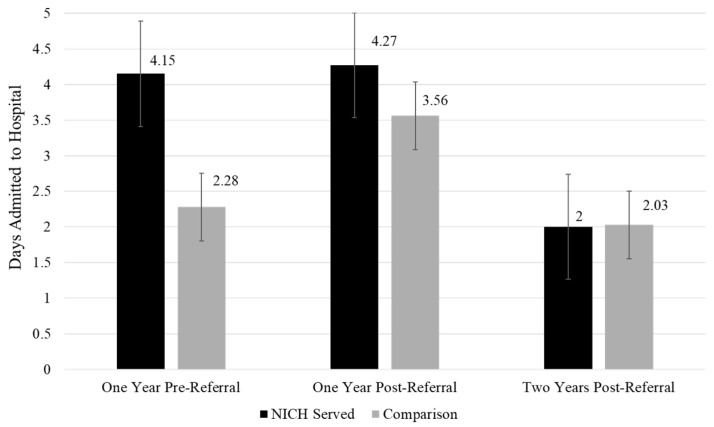
Days admitted across timepoints between groups.

**Figure 4:**
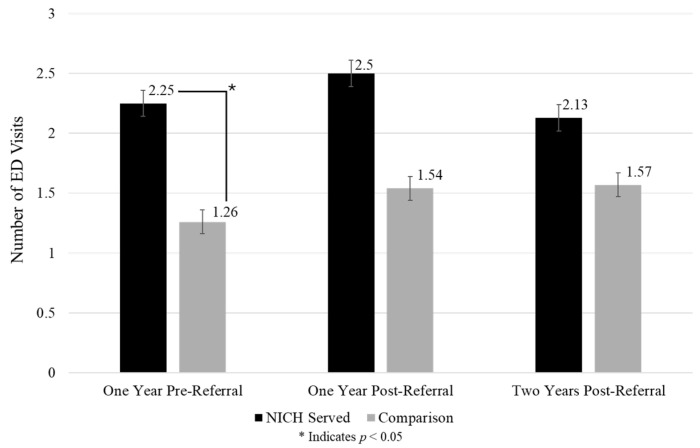
ED visits across timepoints between groups.

Acute event summaries of admissions, days admitted, and ED visits across timepoints and between groups can be found in Figure 2, Figure 3 and Figure 4.

Admissions: NICH youth demonstrated a significant reduction in admissions from one year prior (M = 1.55) to two years post referral (M = 0.70) (*p* = 0.011). In the comparison group, there were no significant changes in admissions over time.

Days admitted: NICH youth demonstrated reductions in days admitted over time that approached significance (*p* = 0.057) from pre-referral (M = 4.15) to two years post-referral (M = 2.00). Comparison youth did not show any significant changes over time in days admitted to the hospital.

ED visits: Neither group demonstrated significant changes in ED visit frequency from pre-referral to one year post-referral (NICH group *p*-value = 0.20; comparison group *p*-value = 0.23) and two years post-referral (NICH group *p*-value = 0.65; comparison group *p*-value = 0.31).

HbA1c: NICH youth demonstrated reduced HbA1c values from pre-referral (M = 11.64%; 287 mg/dL; 15.9 mmol/L) to one year post-referral (M = 10.87%; 265 mg/dL; 14.7 mmol/L) (*p* = 0.006). In comparison youth, there were no significant changes in HbA1c values.

#### 3.2.2. Between-Group Analyses

**Figure 5:**
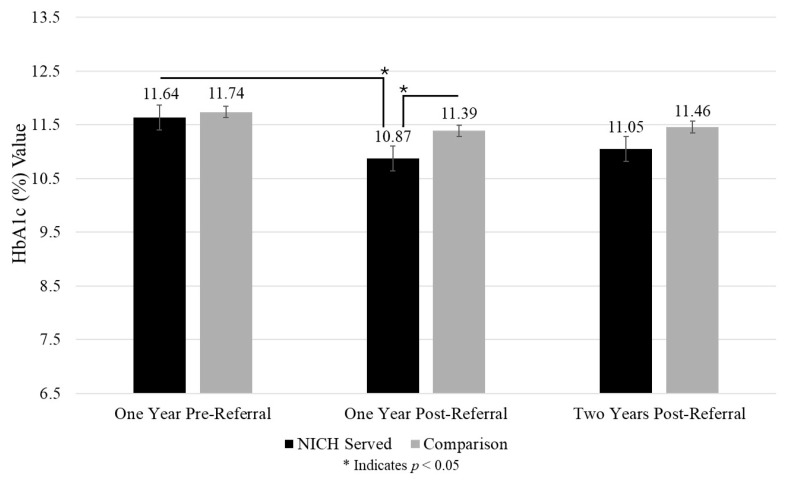
HbA1c (%) across timepoints between groups.

Glycemia: Among youth with chronically high HbA1c values (greater than or equal to 10%), results showed no significant demographic difference between NICH and comparison youth when examining age, gender, race, and ethnicity between groups. NICH youth were significantly less likely to experience an HbA1c over 10% (240 mg/dL; 13.3 mmol/L) (*p* = 0.03) compared to comparison youth at one year post-referral. Results are summarized in Figure 5.

ED visits: There were no significant differences in ED frequency in comparing the NICH group to the comparison group when examining one year post-referral (*p* = 0.07) and two years post-referral (*p* = 0.19).

Admissions: There were no significant differences in admissions in comparing the NICH group to the comparison group when examining one year post-referral (*p* = 0.58) and two years post-referral (*p* = 0.80).

Days: There were no significant differences in days admitted to the hospital when comparing the NICH group to the comparison group when examining one year post-referral (*p* = 0.66) and two years post-referral (*p* = 0.78).

HbA1c: There were no differences in HbA1c values when comparing the NICH group to the comparison group when examining one year post-referral (*p* = 0.20) and two years post-referral (*p* = 0.41).

#### 3.2.3. Repeated Measures ANOVA

**Figure 6:**
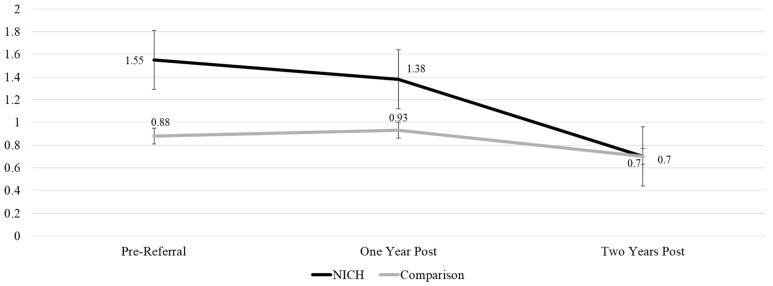
Linear trend in admissions over time for NICH and unserved groups.

Health outcomes (HbA1c, ED visits, admissions, days admitted) were assessed across all three timepoints to compare differences over time between NICH and comparison groups. Results from repeated measure ANOVAs between groups can be seen in Table 2. ANOVA analyses showed a significantly greater reduction in admissions over time for NICH youth compared to comparison youth F (1,95) = 4.036 (*p* = 0.047). This finding can be seen graphically in Figure 6.

**Figure 7:**
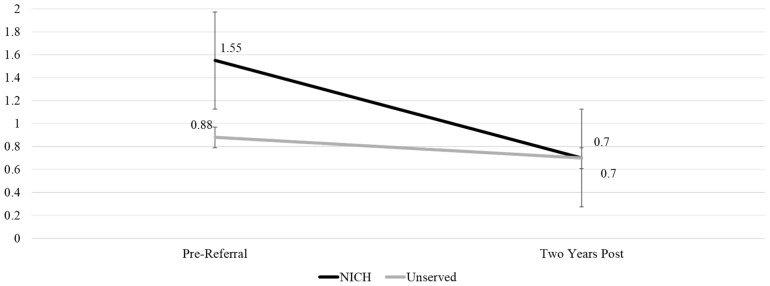
Multivariate comparison of pre-admit and two-year admit totals by NICH group.

We additionally assessed admissions using one year pre- and two years post-NICH referrals as timepoint references. There were significant differences in admissions over time for NICH youth compared to comparison youth (*p* = 0.05). Caution is warranted due to the covariance assumption as indicated by Box’s test of equality of covariance matrices (*p* < 0.001). This finding can be seen visually in Figure 7.

### 3.3. Supplemental Analyses

Given significant differences identified between NICH and comparison groups in key demographic and outcome variables prior to referral, supplementary analyses were conducted using matched pairs. Matching criteria include age at referral, sex, HbA1c, and level of acute utilization, and this process resulted in the identification of 27 pairs of NICH participants and matched comparison youth.

We first assessed the normality of the matched pair data. Timepoint outcomes related to admissions, days admitted, and ED visits were all characterized as violating normality as seen in the significant Shapiro–Wilk test (*p* < 0.001). When examining HbA1c values, pre-referral and two years post-referral violated normality (*p* = 0.01; *p* = 0.23). As such, we utilized nonparametric tests for all analyses except when assessing between-groups for one year post-referral HbA1c changes.

When examining matched pairs (*n* = 54), we identified that there were no significant differences in prior utilization (ED visits, admissions, days admitted) or HbA1c values (*p* > 0.05). There was a significant difference in insurance coverage, with the comparison youth more likely to have private insurance than NICH youth. When running similar tests as noted in the full dataset, we found the below significant findings.

Within-group differences: In NICH youth, there was a significant decrease in HbA1c from pre-referral (M = 11.91%; 295 mg/dL; 16.4 mmol/L; SD = 1.80) to one year post-referral (M = 11.14%; 273 mg/dL; 15.2 mmol/L; SD = 1.84), (*p* = 0.02). While not significant, the NICH group continued to show decreases in ED visits pre-referral (M = 2.04; SD = 1.95) to two years post-referral (M = 1.96; SD = 2.79). There were also non-significant decreases in admissions from pre-referral (M = 1.41; SD = 1.85) to one year post-referral (M = 1.11; SD = 1.31) and to two years post-referral (M = 0.56; SD = 0.80). Lastly, there was a nonsignificant decrease in days admitted to the hospital from one year pre-referral (M = 3.89; SD = 5.54) to two years post-referral (M = 1.41; SD = 2.15). In the comparison group, there were no significant changes across timepoints.

Between-group differences: When examining between-group differences over time, there were no significant differences in outcome variables.

## 4. Discussion

To date, this is the first study assessing the impact of NICH on the health outcomes of under-resourced youth with T1D compared to similarly referred youth. Findings demonstrate that NICH youth showed significant improvements across health outcomes that were not replicated with comparison youth, and that NICH youth showed significantly greater reductions in hospital admissions and lower likelihood of HbA1c elevated values over time compared to comparison youth. Outcomes also add weight to prior findings indicating that prior complications are representative of increased risk of future complications. Results highlight the value of intensive interventions for under-resourced youth.

### 4.1. Limitations

Despite the innovative approach presented in this study, limitations remain. First, this study relied on retrospective chart reviews that likely under-capture health outcomes. While all youth were referred following contact with the home institution’s EHR, only NICH youth were guaranteed to have follow-up labs and utilization available due to their program participation. Thus, comparison youth were at greater risk of experiencing acute utilization in the follow-up period that would not be captured for this study. Second, comparison youth may have engaged in additional treatments that were unable to be captured in this study, potentially impacting their outcomes in ways unknown to this study. Third, findings showing that NICH youth’s time on the waitlist was correlated with health problems in the first year following referral—which is consistent with prior studies demonstrating that NICH youth do not demonstrate improved health between referral and NICH start date—likely bias findings in favor of comparison youth. Lastly, while current NICH programs in Oregon and California serve much more racially and ethnically diverse populations than exist in surrounding communities, the population served in this study was predominantly non-Hispanic white and not fully representative of the broader population of youth with T1D experiencing health inequities.

### 4.2. Strengths

This study had multiple strengths, including following youth across three years, use of objective health data, examination of a population typically understudied in intervention research, and inclusion of youth regardless of family English proficiency and youth developmental level. This study was also the first to evaluate the NICH program outcomes using EHR with a retrospective comparison group to better understand the potential impact of this intervention while attempting to avoid the need for a clinical trial. When considering the level of medical and social vulnerability of participating youth and the potential for critical impacts on health outcomes or even mortality [16,17], a retrospective study design is deemed an appropriate alternative rather than possibly randomizing or delaying services for this group in other research formats [22]. Although retrospective cohort studies may be affected by the limitations of recall bias or selection bias, we believe the use of shared referral needs, primary diagnosis, and presence of social risk for each group reasonably reduces bias risk. Additionally, the data used for evaluation exist in patients’ previously established EHR, eliminating recall bias.

Follow-up studies could improve understanding of NICH impact through methodological modifications. For example, larger sample sizes would improve the interpretability of matched pairs analyses, as some current findings trend toward significance. Furthermore, prospective recruitment of similar populations as well as additional qualitative analyses of patient and family perspectives may capture useful information regarding patient experiences and outcomes. Additionally, future analyses may account for the youths referred to NICH who never received the intervention due to death during time on the waitlist. As previously noted regarding study design, randomization or delaying services among such a vulnerable group raises significant ethical concerns given the risk for mortality or other acute T1D-related concerns that may arise while a youth remains on the waitlist. Furthermore, follow-up studies should include more youth from racial and ethnic groups that have been historically and currently marginalized and discriminated against, which would better reflect the current referral demographic makeup (over 50% Black, Indigenous, and people of color with a variation in ethnic and racial backgrounds depending on the implementation site). Given the disparities experienced by youth from historically marginalized populations, the inclusion of these youth in future research should be a priority. Finally, longer follow-up periods may provide valuable information to the field regarding long-term effects of interventions such as NICH. Prior claims-based findings indicate that the health-related benefits of NICH are often most salient 18 to 24 months following program enrollment, likely due to initial program efforts strongly focused on addressing a multitude of social risks—with disease management being secondary. In addition, the evaluation of NICH in this study includes a time period in which many NICH youth did not receive NICH for the entire period (i.e., one year post-referral). Taken together, it is likely this study under-captures program outcomes due to limitations of the follow-up period.

Overall, both significant findings and health trends from this study highlight the value of NICH in reducing avoidable complications. Although reducing HbA1c is not the primary focus of the NICH intervention, some improvements may result due to overall supports implemented for children with T1D. Even small improvements in HbA1c levels are valuable when considering health outcomes, and a trend of improvement, regardless of statistical significance, may indicate positive momentum toward impactful changes. Notably, comparison youth did not demonstrate changes over time, highlighting how high social risk can lead to chronic health challenges in youth and further escalates the need for healthcare systems to have additional intensive services in their continuum of care. This study adds to the growing literature indicating that behavioral health interventions such as NICH positively impact the health outcomes of vulnerable youth. Youth with complex health and social circumstances are often not well served or included in behavioral health interventions, and NICH creates a valuable and unique opportunity to serve this often-overlooked group. Recognizing that self-management habits developed following NICH enrollment may continue to grow over time, we anticipate the impact of NICH to positively shape the health outcomes of youth experiencing health disparities for years to come. Studies such as this underscore the importance of spreading nationally evidence-based programs that are tailored for those with the greatest need and for healthcare systems and communities to invest in equitable solutions that will benefit both these deserving populations and the broader public. The impacts of NICH indicate the necessity of similar programs that can proactively address the needs of the most vulnerable populations and healthcare inequities on a broader scale. Through research and dissemination efforts locally and nationwide, NICH continues to expand access in the Pacific Northwest, implement the model in new healthcare systems in California, and explore potential new sites across other regions where youth and their families experience similar health and life inequities.

## Figures and Tables

**Table 1:** Frequencies and mean differences for unmatched vs. matched observations.

	Before Matching		After Matching	
	NICH (*n* = 40)	Comparison (*n* = 61)	NICH (*n* = 27)	Comparison (*n* = 27)
Female	23 (57.5%)	28 (45.9%)	15 (55.6%)	15 (55.6%)
White	34 (85.0%)	53 (86.9%)	24 (88.9%)	25 (92.6%)
Multiracial	3 (7.5%)	3 (4.9%)	1 (3.7%)	1 (3.7%)
African American/Black	2 (5.0%)	1 (1.6%)	2 (7.4%)	0 (0.0%)
Pacific Islander	1 (2.5%)	1 (1.6%)	0 (0.0%)	1 (3.7%)
Asian	0 (0.0%)	1 (1.6%)	0 (0.0%)	0 (0.0%)
Hispanic	5 (12.5%)	7 (11.5%)	2 (7.4%)	3 (11.1%)
Mean age at referral (SD)	14.11 (3.36)	14.01 (3.43)	14.35 (1.84)	14.44 (1.77)
Means of One Year Pre-Referral Medical Utilization (SD)				
Admissions	1.55 (1.73)	0.89 (0.92)	1.41 (1.85)	1.19 (1.04)
Days admitted	4.15 (5.04)	2.28 (2.41)	3.89 (5.54)	2.81 (2.39)
ED visits	2.25 (2.22)	1.26 (1.21)	2.04 (1.95)	1.56 (1.34)
HbA1c (%)	11.64 (1.84)	11.74 (1.76)	11.91 (1.80)	11.67 (1.62)

**Table 2:** NICH program impacts across different timepoints.

Outcome Means (SD)	NICH 1 Year Pre-Referral	Comparison 1 Year Pre-Referral	NICH 1 Year Post-Referral	Comparison 1 Year Post-Referral	NICH 2 Years Post-Referral	Comparison 2 Years Post-Referral	*p*-Value
Admissions	1.55 (1.72)	0.89 (0.92)	1.38 (2.08)	0.95 (1.29)	0.70 (0.99)	0.70 (1.16)	0.11
Days Admitted	4.15 (5.03)	2.28 (2.40)	4.27 (6.55)	3.56 (6.42)	2.00 (3.07)	2.03 (3.88)	0.17
ED visits	2.25 (2.21)	1.26 (1.21)	2.50 (2.70)	1.54 (1.88)	2.13 (2.56)	1.57 (2.10)	0.73
HbA1c (%)	11.64 (1.84)	11.74 (1.76)	10.87 (1.91)	11.39 (2.12)	11.05 (2.09)	11.46 (1.96)	0.17

## Data Availability

Please make data requests to Dr. Michael Harris at harrismi@ohsu.edu.

## References

[1] Wagner D.V., Barry S.A., Stoeckel M., Teplitsky L., Harris M.A. (2017). NICH at its best for diabetes at its worst: Texting teens and their caregivers for better outcomes. J. Diabetes Sci. Technol..

[2] Borus J.S., Laffel L. (2010). Adherence challenges in the management of type 1 diabetes in adolescents: Prevention and intervention. Curr. Opin. Pediatr..

[3] Keenan H.T., Foster C.M., Bratton S.L. (2002). Social factors associated with prolonged hospitalization among diabetic children. Pediatrics.

[4] Feldman M.A., Anderson L.M., Shapiro J.B., Jedraszko A.M., Evans M., Weil L.E.G., Garza K.P., Weissberg-Benchell J. (2018). Family-based interventions targeting improvements in health and family outcomes of children and adolescents with type 1 diabetes: A systematic review. Curr. Diab Rep..

[5] Duke D.C., Wagner D.V., Ulrich J., Freeman K.A., Harris M.A. (2016). Videoconferencing for teens with diabetes: Family matters. J. Diabetes Sci. Technol..

[6] George S., Duran N., Norris K. (2014). A systematic review of barriers and facilitators to minority research participation among African Americans, Latinos, Asian Americans, and Pacific Islanders. Am. J. Public Health.

[7] Lehmkuhl H.D., Storch E.A., Cammarata C., Meyer K., Rahman O., Silverstein J., Malasanos T., Geffken G. (2010). Telehealth behavior therapy for the management of type 1 diabetes in adolescents. J. Diabetes Sci. Technol..

[8] Agarwal S., Kanapka L.G., Raymond J.K., Walker A., Gerard-Gonzalez A., Kruger D., Redondo M.J., Rickels M.R., Shah V.N., Butler A. (2020). Racial-ethnic inequity in young adults with type 1 diabetes. J. Clin. Endocrinol. Metab..

[9] Agarwal S., Hilliard M., Butler A. (2018). Disparities in care delivery and outcomes in young adults with diabetes. Curr. Diab. Rep..

[10] Harris M.A. (2018). Your exclusion, my inclusion: Reflections on a career working with the most challenging and vulnerable in diabetes. Diabetes Spectr..

[11] Harris M.A., Spiro K., Heywood M., Wagner D.V., Hoehn D., Hatten A. (2013). Novel interventions in children’s health care (NICH): Innovative treatment for youth with complex medical conditions. Clin. Pract. Pediatr. Psychol..

[12] Harris M.A., Heywood M., Wagner D.V., Hoehn D., Bahia H., Spiro K. (2014). Youth repeatedly hospitalized for DKA: Proof of concept for Novel Interventions in Children’s Healthcare (NICH). Diabetes Care.

[13] Shrestha S.S., Zhang P., Barker L., Imperatore I. (2010). Medical expenditures associated with diabetes acute complications in privately insured U.S. youth. Diabetes Care.

[14] Wysocki T., Harris M.A., Buckloh L.M., Mertlich D., Sobel Lochrie A., Taylor A., Sadler M., Mauras N., White N.H. (2005). Effects of behavioral family systems therapy for diabetes on adolescents’ family relationships, treatment adherence, and metabolic control. J. Pediatr. Psychol..

[15] Harris M.A., Wagner D.V., Dukhovny D. (2016). Commentary: Demon$trating (our) value. J. Pediatr. Psychol..

[16] Ellison G.W., Mickey R.M., Myers L.W. (1988). Alternatives to randomized clinical trials. Neurology.

[17] Rundgren S., Brus O., Båve U., Landén M., Lundberg J., Nordanskog P., Nordenskjöld A. (2018). Improvement of postpartum depression and psychosis after electroconvulsive therapy: A population-based study with a matched comparison group. J. Affect. Disord..

[18] Sedgwick P. (2014). Retrospective cohort studies: Advantages and disadvantages. BMJ.

[19] Shim M.J., Lee Y.S., Oh H.E., Kim J.S. (2007). Effects of a back-pain-reducing program during pregnancy for Korean women: A non-equivalent control-group pretest–posttest study. Int. J. Nurs. Stud..

[20] Dearing M.E., Bowles S., Isenor J., Kits O., O’Donnell L.K., Neville H., Hilmer S., Toombs K., Sirois C., Hajzadeh M. (2020). Pharmacist-led intervention to improve medication use in older inpatients using the Drug Burden Index: A study protocol for a before/after intervention with a retrospective control group and multiple case analysis. BMJ Open.

[21] Nundy S., Cooper L.A., Mate K.S. (2022). The quintuple aim for health care improvement: A new imperative to advance health equity. JAMA.

[22] Kim J., Choi S.M., Lee J., Park Y.S., Lee C.H., Yim J.J., Yoo C.G., Kim Y.W., Han S.K., Lee S.M. (2017). Effect of renin-angiotensin system blockage in patients with acute respiratory distress syndrome: A retrospective case control study. Korean J. Crit. Care Med..

